# Supplementary material for: A mechanism of cohesin‐dependent loop extrusion organizes zygotic genome architecture
Source: EMBO J. 2017 Dec 7;36(24):3600–18. doi: 10.15252/embj.201798083 (PMC5730859; doi:10.15252/embj.201798083)
Supplement: Supplementary file 1 — Appendix [file EMBJ-36-3600-s001.pdf]

Appendix Table of Contents:

Appendix Figure S1……………………………………………………………………………………p.2-3

Appendix Figure S2……………………………………………………………………………………p.4-5

Appendix Figure S3……………………………………………………………………………………p.6-7

Appendix Figure S4……………………………………………………………………………………p.8-9

Appendix Figure S5……………………………………………………………………………………p.10-11

Appendix Figure S6……………………………………………………………………………………p.12-13

Appendix Figure S7……………………………………………………………………………………p.14-15

Appendix Figure S8……………………………………………………………………………………p.16-17

Appendix Figure S9……………………………………………………………………………………p.18-19

Appendix Materials and Methods……………………………………………………………………p.20-21

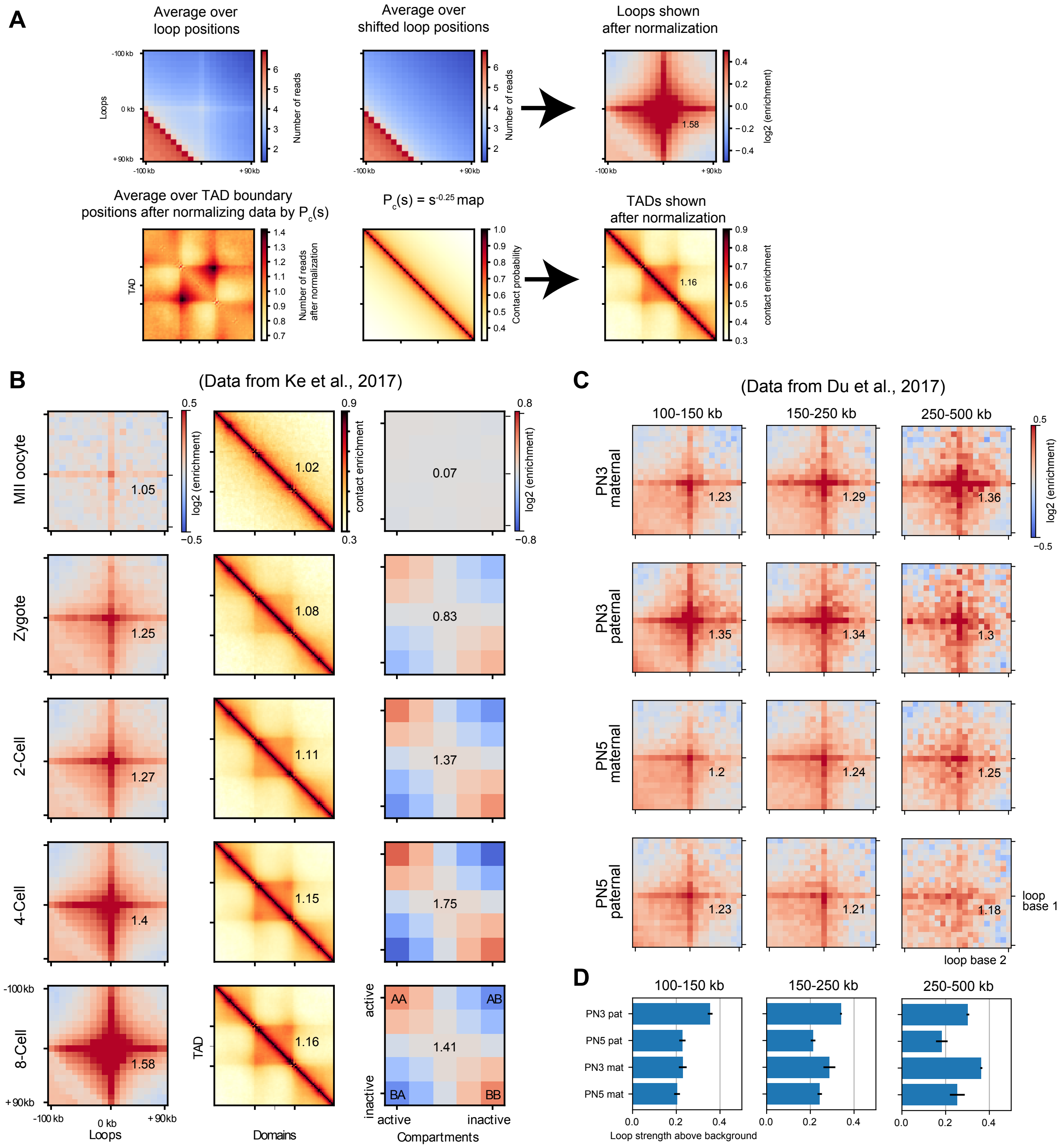

Appendix Figure S1

Appendix Figure S1: Zygotic paternal chromatin has stronger loops

A. The procedure for generating average Hi-C loops and average TADs is demonstrated. For all annotated loops, the raw snHi-C map values are averaged using a 190 kb window centered on the loop base (top left panel); similarly, raw snHi-C map values are averaged using randomly shifted loop positions (top middle panel) to control for contact probability decay with distance. The average loop and shifted positions are then normalized (top right panel) as in Flyamer et al., 2017. For each annotated TAD, a window of 3 times the TAD length was centered on the TAD and contact probability normalized snHi-C map values in this window are re-binned to make a 90 by 90 pixel matrix; all coarse-grained TAD matrices were averaged to produce the average TADs picture (bottom left panel); a shallow contact probability matrix is generated (bottom middle panel) by which the average TAD picture is normalized to help visualize the TAD resulting in the final average TAD picture (bottom right panel) (see **Materials and Methods**).

B. Average Hi-C loops, TADs and compartment saddle plots were generated from bulk oocyte, zygote and embryo Hi-C data (Ke et al., 2017). Loops and TADs were averaged over positions annotated in CH12-LX positions in Rao et al., 2014.

C. Average Hi-C loops, separated by genomic distance computed from bulk zygote Hi-C data (Du et al., 2017). Zygotic pronuclear stage 3 (PN3) and stage 5 correspond to S and G2 phases, respectively. The numeric values in each plot correspond to the fold enrichment in loop strength above background levels. Windows shown are a 190 kb region centered on the loop bases.

D. Quantification of loop strength above background. Reported values are the fraction of enrichment above background. Error bars displayed are the 95% confidence intervals obtained by bootstrapping the experimental replicates (see **Materials and Methods**).

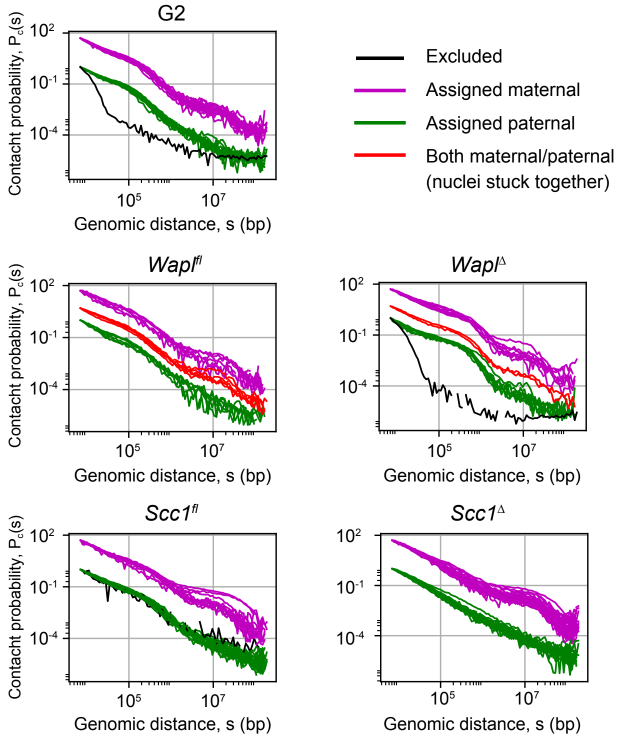

Appendix Figure S2

Appendix Figure S2: Identifying maternal and paternal pronuclei

Contact probability curves were plotted for all snHi-C data to help identify the parent of origin based on the shape of the $P_c(s)$ curve at the 10-30 Mb range. Maternally derived chromatin is characterized by a plateau in contact probability at the 10-30 Mb range (magenta curves), whereas paternally derived chromatin does not exhibit this plateau (green curves). For details on how maternal/paternal nuclei were assigned, see **Methods and Materials**. For nuclei that stuck together during the isolation procedure, we did not assign a maternal/paternal value as these are the average of two distinct chromatin conformations, but they were used for analyses which did not account for parental source. Contact probability curves for maternal/paternal/both are vertically shifted in order to more easily see the differences in shape.

2D projection of cohesin locations from a representative conformation of the simulation (X- and Y- axis units in monomers, 1 monomer = 15 nm)

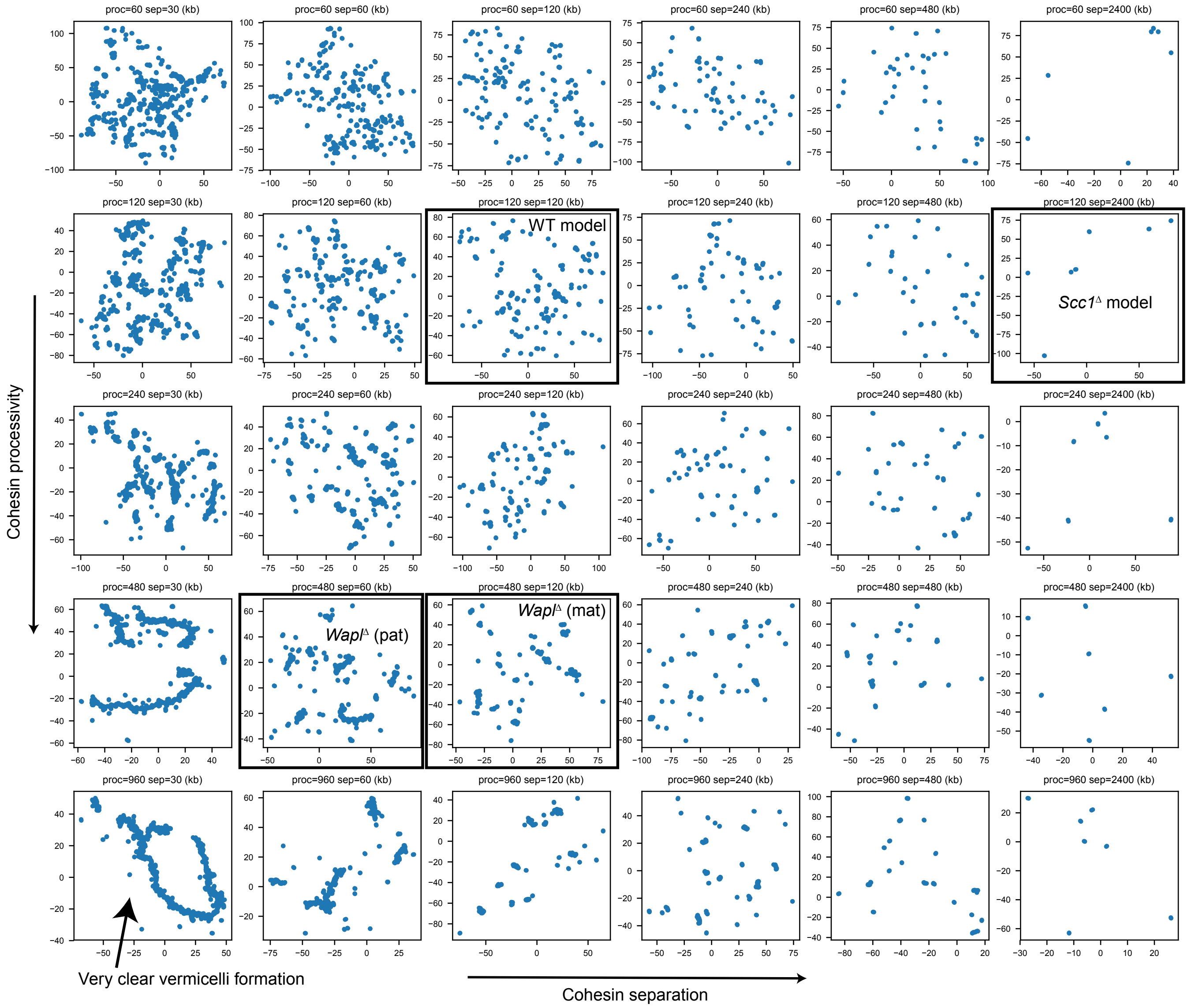

Appendix Figure S3

Appendix Figure S3: Cohesin stains from polymer simulations of the loop extrusion mechanism

Two-dimensional projections of cohesin locations are shown for representative chromosome conformations. The table is organized to correspond to **Figure EV 4A** which shows the slope of the log $P_c(s)$ curves. Of note, as cohesin processivity (proc) increases and separation (sep) decreases, long strings of cohesins form due to crowding of cohesins as they bump into each other and stall. We posit that these long strings form the basis of cohesin enriched axial structures termed "vermicelli" that have been described previously in *Wapl*$^{\Delta}$ cells (Tedeschi et al., 2013; Lopez-Serra et al., 2013; Haarhuis et al., 2017).

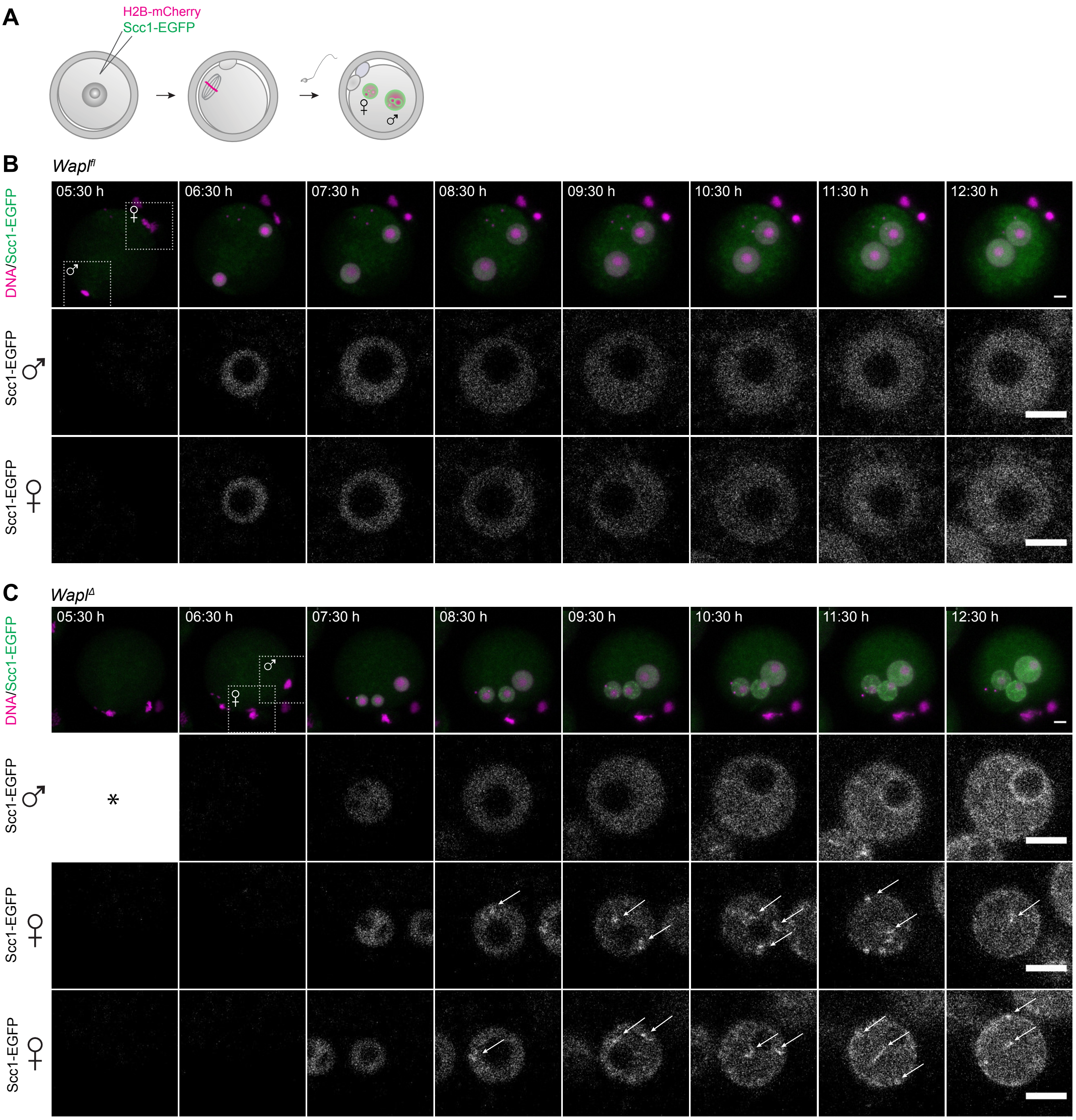

Appendix Figure S4

Appendix Figure S4: Live-cell imaging of vermicelli formation in *Wapl*^{fl} and *Wapl*$^{\Delta}$ zygotes expressing Scc1-EGFP and H2B-mCherry

A. Germinal vesicle-stage oocytes were injected with mRNA encoding H2B-mCherry to mark chromosomes (magenta) and Scc1-EGFP to label cohesin (green), matured to meiosis II, fertilized *in vitro* and followed by time-lapse microscopy.

B. Still images of live *Wapl*^{fl} zygotes expressing Scc1-EGFP and H2B-mCherry (n=2 zygotes, from one experiment using two females). Top row: Z-stack maximum intensity projection of zygotes. Middle and bottom row: Z-slices of the cropped areas showing paternal and maternal nuclei separately. Scale bar: 10µm.

C. Still images of live *Wapl*$^{\Delta}$ zygotes expressing Scc1-EGFP and H2B-mCherry (n=1, from one experiment using two females). Note that this zygote contains one paternal and two maternal nuclei, likely due to failure of cytokinesis in meiosis II. Top row: Z-stack maximum intensity projection of zygotes. Middle and bottom row: Z-slices of the cropped areas showing paternal and two maternal nuclei separately. Arrows indicate Scc1-EGFP enriched structures. The panel for the paternal nucleus at the first time point is missing since it was not captured in the z-stack (indicated by asterisk). Images were adjusted in brightness/contrast in individual imaging channels in the same manner for B) and C). Scale bar: 10 µm. Hours after start of IVF are given.

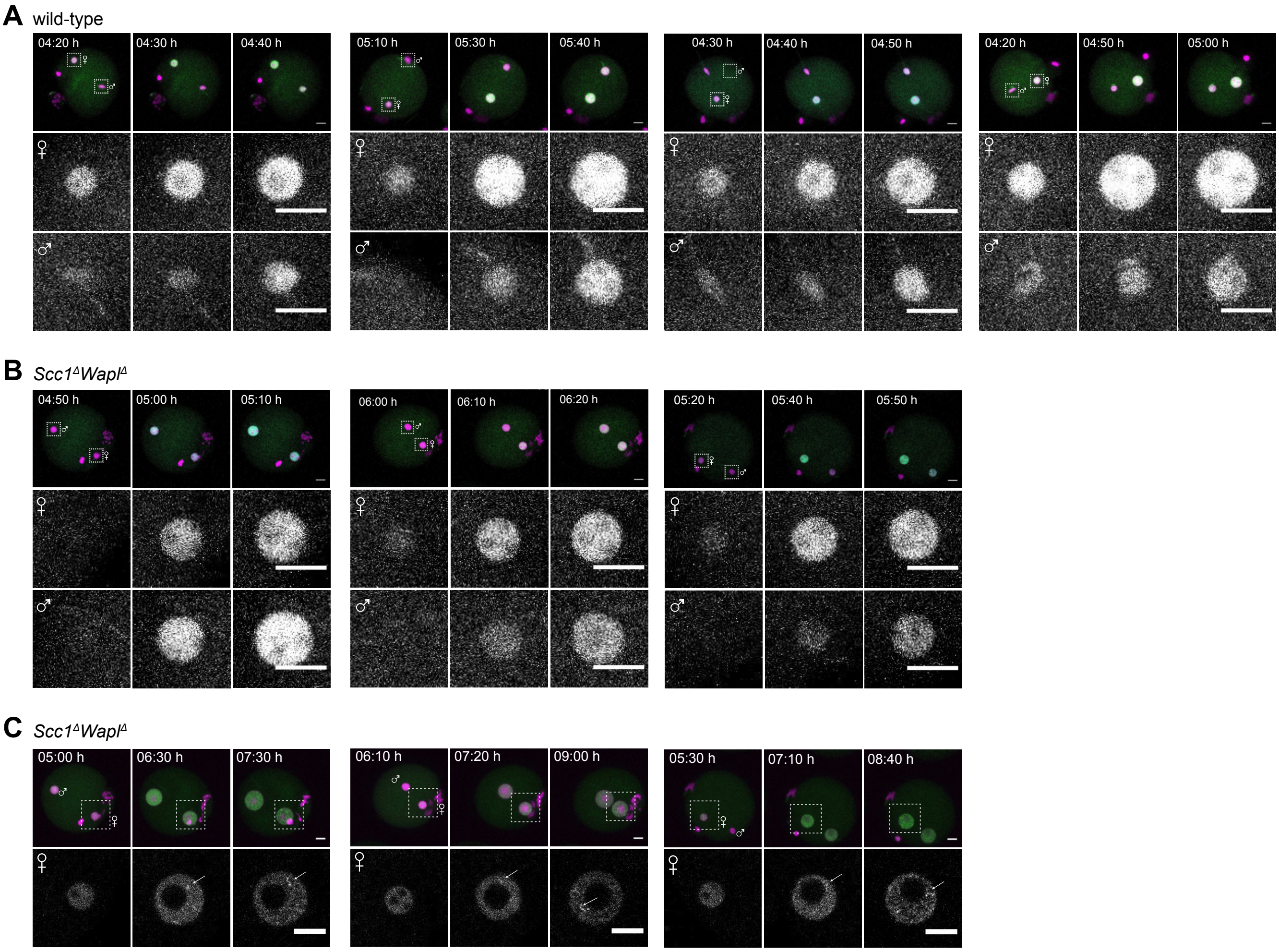

Appendix Figure S5

Appendix Figure S5: Live-cell imaging of wildtype and *Scc1^Δ^Wapl^Δ^* zygotes expressing Scc1-EGFP and H2B-mCherry

A. Onset of Scc1-EGFP accumulation in nuclei of wildtype zygotes (n=4 zygotes, from one experiment using two females). Top row: Z-stack maximum intensity projection of whole zygotes. Middle and bottom row: Z-stack maximum intensity projection of maternal and paternal nuclei.

B. Onset of Scc1-EGFP accumulation in nuclei of *Scc1^Δ^Wapl^Δ^* zygotes (n=3 zygotes, from one experiment using two females). Top row: Z-stack maximum intensity projection of whole zygotes. Middle and bottom row: Z-stack maximum intensity projection of maternal and paternal nuclei.

A. Onset of vermicelli formation in Scc1ΔWaplΔ zygotes (n=3 zygotes, from one experiment using two females) corresponding to B. Top: Z-stack maximum intensity projection of whole zygotes. Bottom: Single z-slices of maternal nuclei.

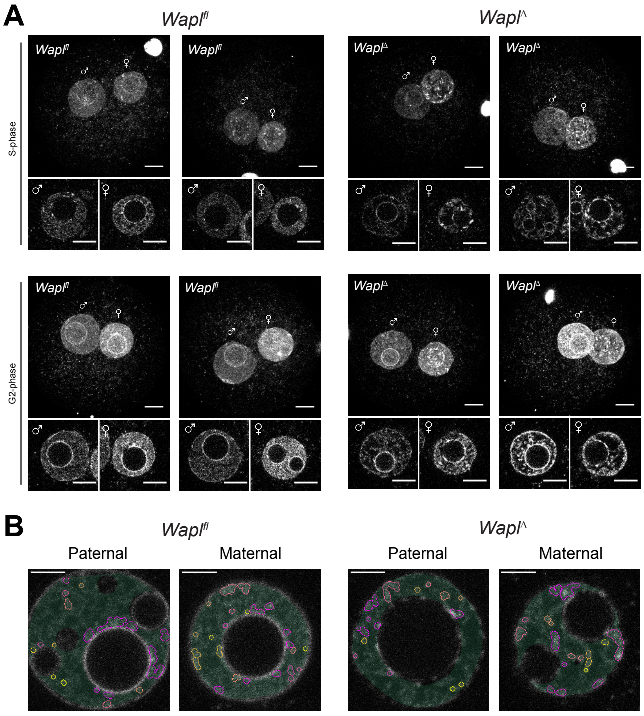

Appendix Figure S6

Appendix Figure S6: DNA staining of *Wapl*^{fl} and *Wapl*$^{\Delta}$ zygotes

A. Representative images of fixed *Wapl*^{fl} and *Wapl*$^{\Delta}$ zygotes stained with DAPI. Zygotes were collected during S phase (9 h 45 min post fertilization; n=7 *Wapl*^{fl}, n=15 *Wapl*$^{\Delta}$) or G2 phase (14 h post-fertilization; n=3 *Wapl*^{fl}, n=8 *Wapl*$^{\Delta}$*;* one experiment using two females per genotype). Top: Z-stack maximum intensity projection (MIP) of zygotes. Bottom: Individual maternal and paternal nuclei. Settings were adjusted for z-slices and MIPs individually, but in the same manner for *Wapl*^{fl} and *Wapl*$^{\Delta}$ zygotes. Scale bar: 10µm.

B. Example segmentation masks used in DAPI texture analysis superimposed on original images, together with DAPI-intense structures detected inside the nuclei.

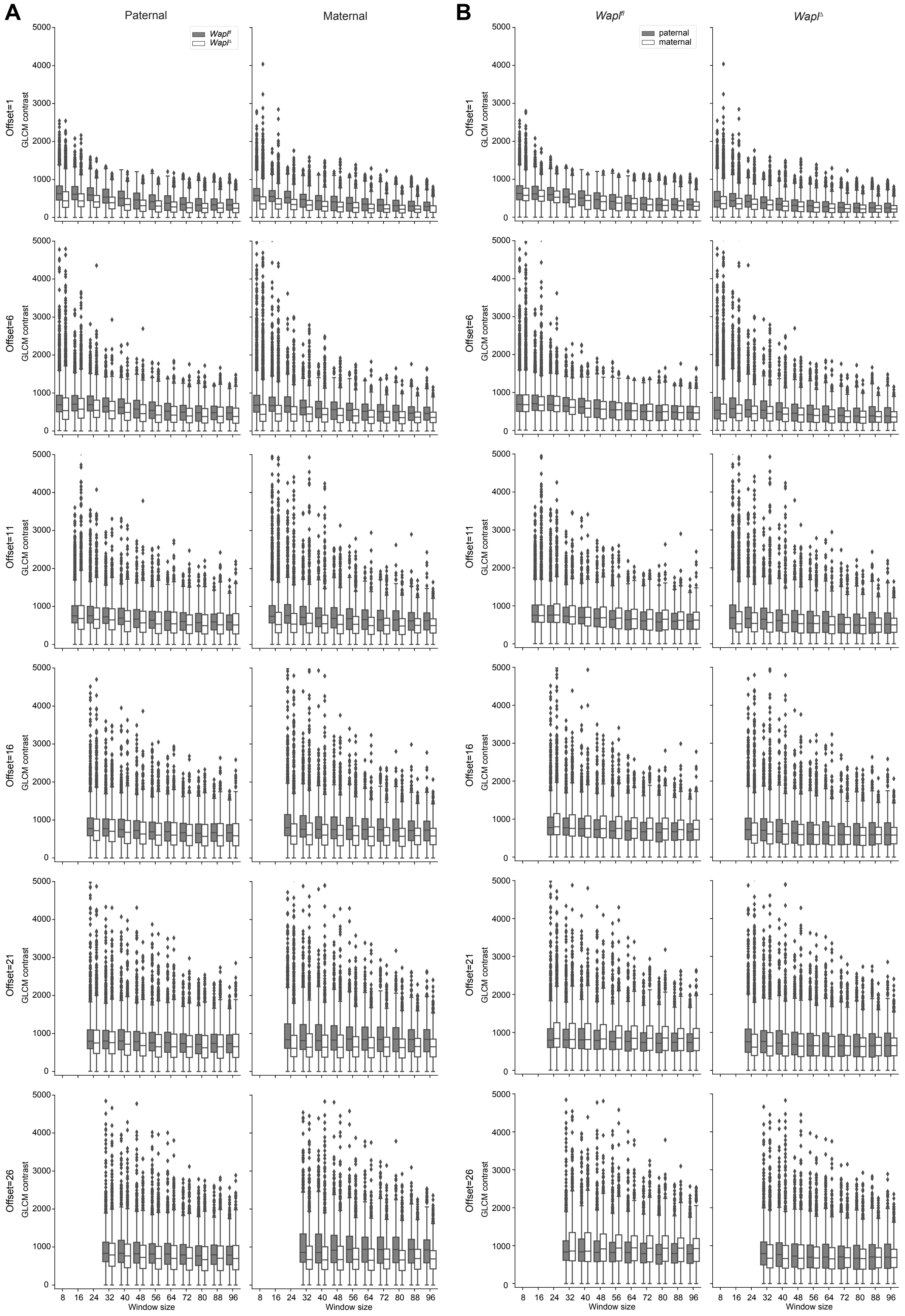

Appendix Figure S7

Appendix Figure S7: GLCM contrast analysis of DAPI texture in *Wapl*^{fl} and *Wapl*$^{\Delta}$ zygotes

A. Boxplots showing GLCM contrast (local variation of intensity) in paternal (grey) and maternal (white) nuclei of *Wapl*^{fl} (grey, n=15) and *Wapl*$^{\Delta}$ (white, n=21) zygotes with increasing window sizes and different offset.

B. Boxplots showing GLCM contrast (local variation of intensity) in paternal and maternal nuclei of *Wapl*^{fl} (grey, n=15) and *Wapl*$^{\Delta}$ (white, n=21) zygotes with increasing window sizes and different offset. (Data from two independent experiments using two females per genotype.)

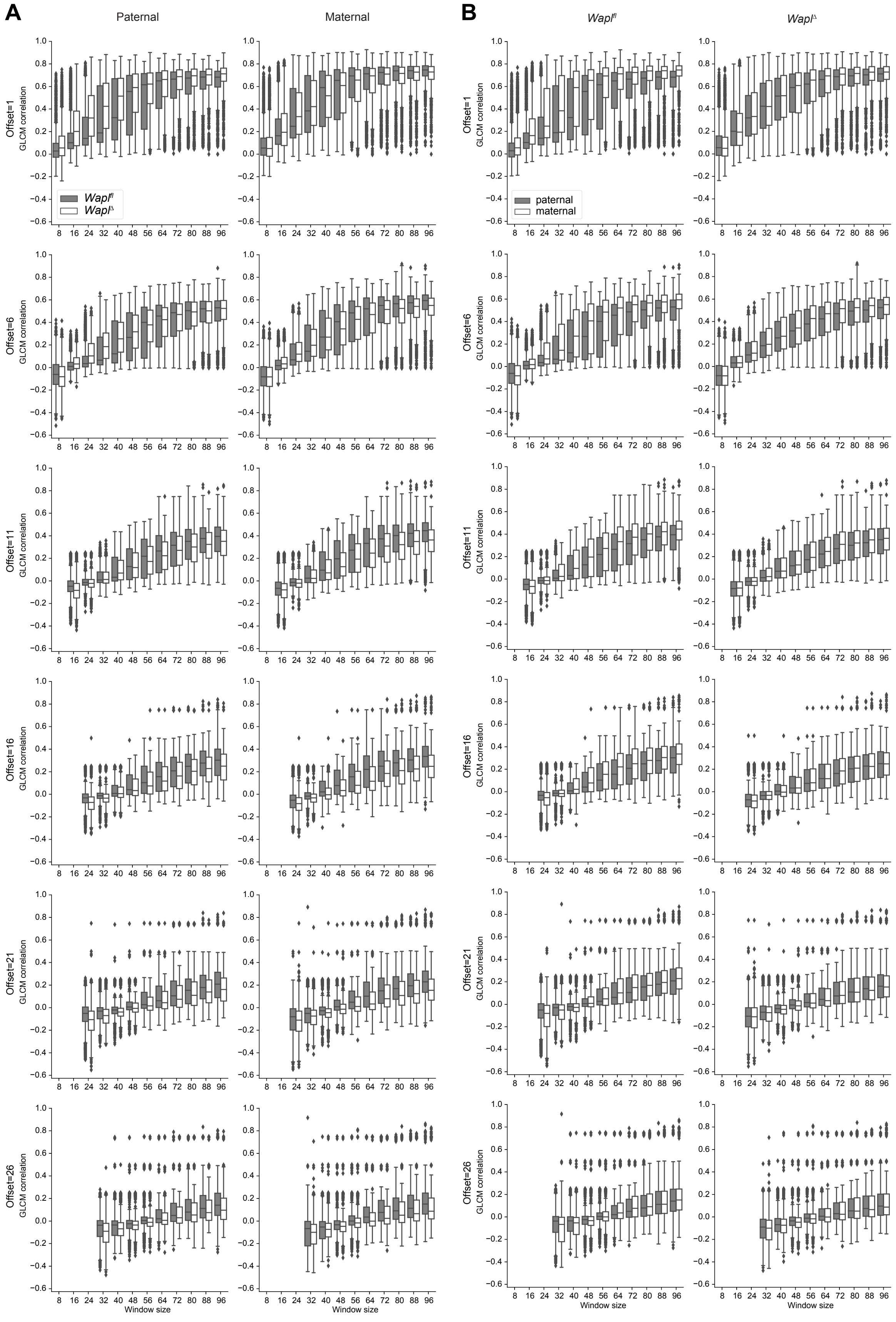

Appendix Figure S8

Appendix Figure S8: GLCM correlation analysis of DAPI texture in $Wapl^{fl}$ and $Wapl^{\Delta}$ zygotes

B. Boxplots showing GLCM correlation (linear dependence of intensity between pixels) in paternal and maternal nuclei of *Wapl*^{fl} (grey, n=15) and *Wapl*$^{\Delta}$ (white, n=21) zygotes with increasing window sizes and different offset.

C. Boxplots showing GLCM correlation (linear dependence of intensity between adjacent pixels) in paternal (grey) and maternal (white) nuclei in *Wapl*^{fl} (n=15) and *Wapl*$^{\Delta}$ (n=21) zygotes with increasing window sizes and different offset. (Data from two independent experiments using two females per genotype.)

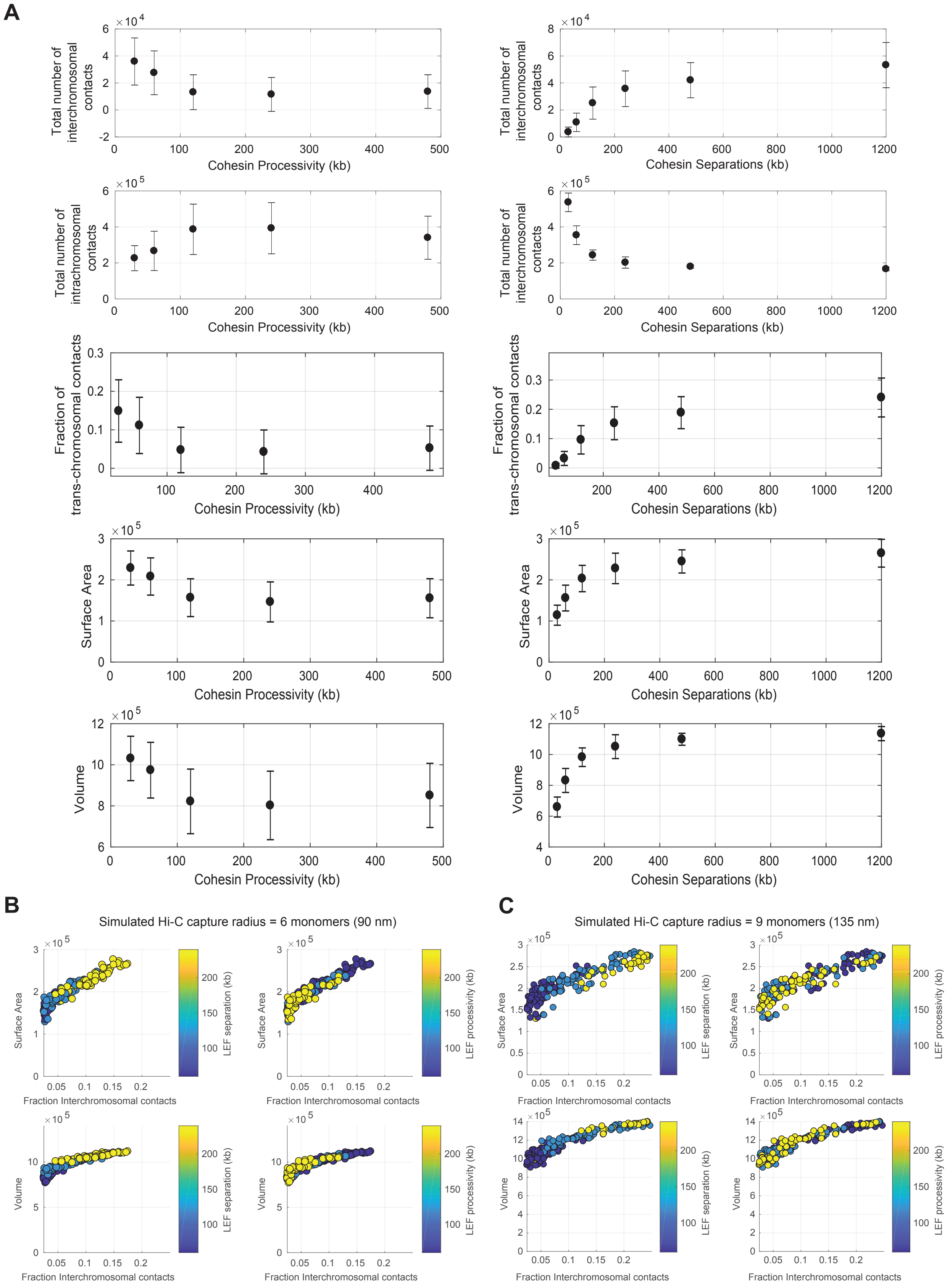

Appendix Figure S9

Appendix Figure S9: Variation of simulation parameters and its effect on surface area, volume, and contact frequencies

A. Volumes, surface area, and the fraction of inter-chromosomal and intra-chromosomal contacts as a function of loop extrusion simulation parameters: cohesin processivity and cohesin separation. Values reported are the averages and standard deviations for each parameter. Averages were computed from three randomly sampled simulation conformations and over all possible simulation parameters tested (See **Materials and Methods**).

B. Surface area was computed using a radius of 90 nm (equivalent to 6 monomer radii), and a simulated Hi-C capture radius of the same value. The fraction of inter-chromosomal contacts was computed without excluding contacts occurring from "self-contacts" due to monomer-monomer interactions separated by less than 6 monomers along the linear genome. This control illustrates that the surface area, volume and *trans*-interaction fractions trend persist even if we change how *trans*-interaction frequencies are defined.

C. Surface area was computed using a radius of 135 nm (equivalent to 9 monomer radii), and a simulated Hi-C capture radius of the same value. The fraction of inter-chromosomal contacts was computed as per usual, by excluding contacts occurring from "self-contacts" (i.e. due to monomer-monomer interactions separated by less than 9 monomers along the linear genome). This control demonstrates that even by increasing the simulated Hi-C capture radius, the general trend of linearity of surface area versus *trans*-contacts, and changes in volume persists.

APPENDIX MATERIALS AND METHODS

DAPI texture analysis

All analysis was performed using python (3.5.2) scientific stack (numpy-1.13.3, scipy-0.19.1, pandas-0.20.2, matplotlib-2.1.0) with image analysis specific functions from scikit-image-0.13.0. Pixel intensities in images of zygotes stained with DAPI were clipped at 99th percentile, then automatically thresholded plane-by-plane using the Otsu method after total variance denoising (denoise_tv_chambolle) with weight 0.2. After binary closing using a disk structuring element with radius 3 and removal of small holes (<50 pixels) and objects (<100 pixels), elongated (major axis length >1.25 times longer than minor axis) and misshapen (circularity below 0.5) holes were also removed. Then objects with area below 15,000 pixels (after filling holes) were removes, and a median filter with a disk structuring element with radius 20 was applied. Then large objects (above 150,000 pixels) were removed, along with dim (with average intensity below 1.5 times average intensity of the whole z-plane) and misshapen (circularity below 0.35) objects. After that all zero values present after filtering of holes were restored. Then all planes were combined to form a single object annotation for the whole z-stack, and again small (<500,000 pixels in volume) objects were removed, and the whole image was labelled with connectivity=1.

After that the segmented images were processed to split touching pronuclei using convexity defects. Again, this was performed for each z-plane separately. First, the z-plane was labelled. If there was more than one object present, or only one but with low (<1.2) ratio of major and minor axes), nothing more was done to this image. Otherwise, a median filter with a disk structuring element with radius 25 was applied, and then binary closing with the same structuring element. After that the holes were filled and convex hull of the object was calculated. Then distance from each pixel in the convex hull outside of the object to the convex hull's edge was calculated, and coordinates of local peaks of these distances were found with a minimal distance of 20 between them and a minimal absolute threshold of 15. If at least two peaks were found, the two highest of them were taken, and their coordinates were recorded. In case there were no or only one convexity defect found, but the object was elongated (ratio of major and minor axes >1.3), the object was removed. After this was done to the whole z-stack, coordinates of convexity defects were used to separate touching objects. To do this a line of zeros with width of 15 pixels connecting the coordinates was drawn for each pair of convexity defects in the corresponding z-planes, and 3 planes above and below. Then all planes were processed to remove small (<5,000 pixels) or misshapen (circularity<0.35) objects. Then all holes were dilated by two z-planes in each direction, and by 10 pixels in width, and the whole object was eroded with the same parameters to remove heterochromatin from the segmentation. After removal of small objects (<300,000 pixels) the objects in the whole image were labelled and saved and manually checked for high segmentation quality. The maternal and paternal origin of pronuclei was determined using their size: we considered paternal the ones where both the total volume and the biggest cross-section area were higher than in the other pronucleus, which we then considered maternal; we didn't use images where these two measurements disagreed.

For the coefficient of variation (CV) analysis, all pixels from each object the segmented image were used to calculate mean and standard deviation. For the Gray-Level Co-Occurrence Matrix (GLCM) analysis, we randomly generated 100 2D windows for each image, so that they are fully inside the segmented area, and moreover, so that if their size was increased by 14 pixels along x and y axis and they were shifted up or down by one z plane, they were still fully inside the masked region. We performed this for windows of sizes between 8×8 and 96×96 pixels, with each step increasing the sides of the windows by 8 pixels, in total 1200 windows per nucleus. We then applied GLCM analysis of correlation and contrast to each of the windows. The GLCM matrices were constructed for 4 directions, and for offsets between 1 and 26 with step 5 (only offsets smaller than the window size were used). Contrast and correlation measures were calculated for each of these conditions, but then

averaged across 4 directions for each offset value. Data from all windows were combined together, recording their origin. When plotting contrast, the y axis limit was set to 5000, and any outliers above that are not shown. For the analysis of bright objects, segmented nuclei were thresholded using Otsu's method, then median filtering and binary opening (using a disk structuring element with radius 3) were applied to each z-plane of the image, and the objects were labelled in 3D using connectivity=1. Number of pixels in each object was taken as their volume. All P-values were calculated using the Mann-Whitney U test. Cells where nuclei were not properly segmented or separated, or at least one nucleolus was not excluded were not used in the analysis.

Polymer simulation surface area and volume measurements

We calculated the surface area and volume of single polymer conformations using the MATLAB R2017a alphaShapes class. In brief, 3 polymer conformations were randomly sampled for each simulated chromatin condition from the system of 30,000 monomer simulated chromatin fibers. For a given configuration, we calculated the numbers of contacts in *cis* and *trans* using a cutoff radius of 5 monomer radii. *Trans* contacts were computed from the contacts of the polymer with its 26 periodic boundary images in the neighbouring simulation volumes. To calculate the surface area and volume, we defined spheres of radius equal to the Hi-C capture frequency (unless otherwise noted) around each monomer using the "sphere" function, with input argument 10, and computed the alpha shape on the resulting set of points with alpha parameter 1.6 to account for variable bond distances between monomers due to the harmonic potential. The surface area and volumes were computed using the .surfaceArea and .volume methods respectively. Results of the polymer simulations were plotted against the calculated number of *cis* and *trans* contacts.
