# Supplementary material for: A mechanism of cohesin‐dependent loop extrusion organizes zygotic genome architecture
Source: EMBO J. 2017 Dec 7;36(24):3600–18. doi: 10.15252/embj.201798083 (PMC5730859; doi:10.15252/embj.201798083)
Supplement: Supplementary file 2 — Expanded View Figures PDF [file EMBJ-36-3600-s002.pdf]

Expanded View Figures

Figure EV1. Average TADs called in many cell types show enrichments in zygote Hi-C and snHi-C.

A TAD boundaries were *de novo* identified in many cell types and Hi-C data sets using the corner score as described in Schwarzer *et al* (2017) using the lavaburst software. The *de novo* identified boundaries were used to generate average TAD profiles (shown in B), and the TAD strength was computed (see Materials and Methods). Notably, all data sets showed enrichments for TADs for all identified boundaries except MII oocytes (Du *et al*, 2017) which are in mitosis and are not expected to have TADs (Naumova *et al*, 2013), and our *Scc1*$^{\Delta}$ zygotes. All TAD strength analyses for snHi-C data sets are highlighted (purple box).

B Average TAD profiles in different data sets are shown called from boundaries identified in mouse ES cells (Nora *et al*, 2017) using the corner score as in Schwarzer *et al* (2017). These average TAD profiles were used to calculate the TAD strengths in (A) for the top row of the matrix.

Source data are available online for this figure.

© 2017 The Authors

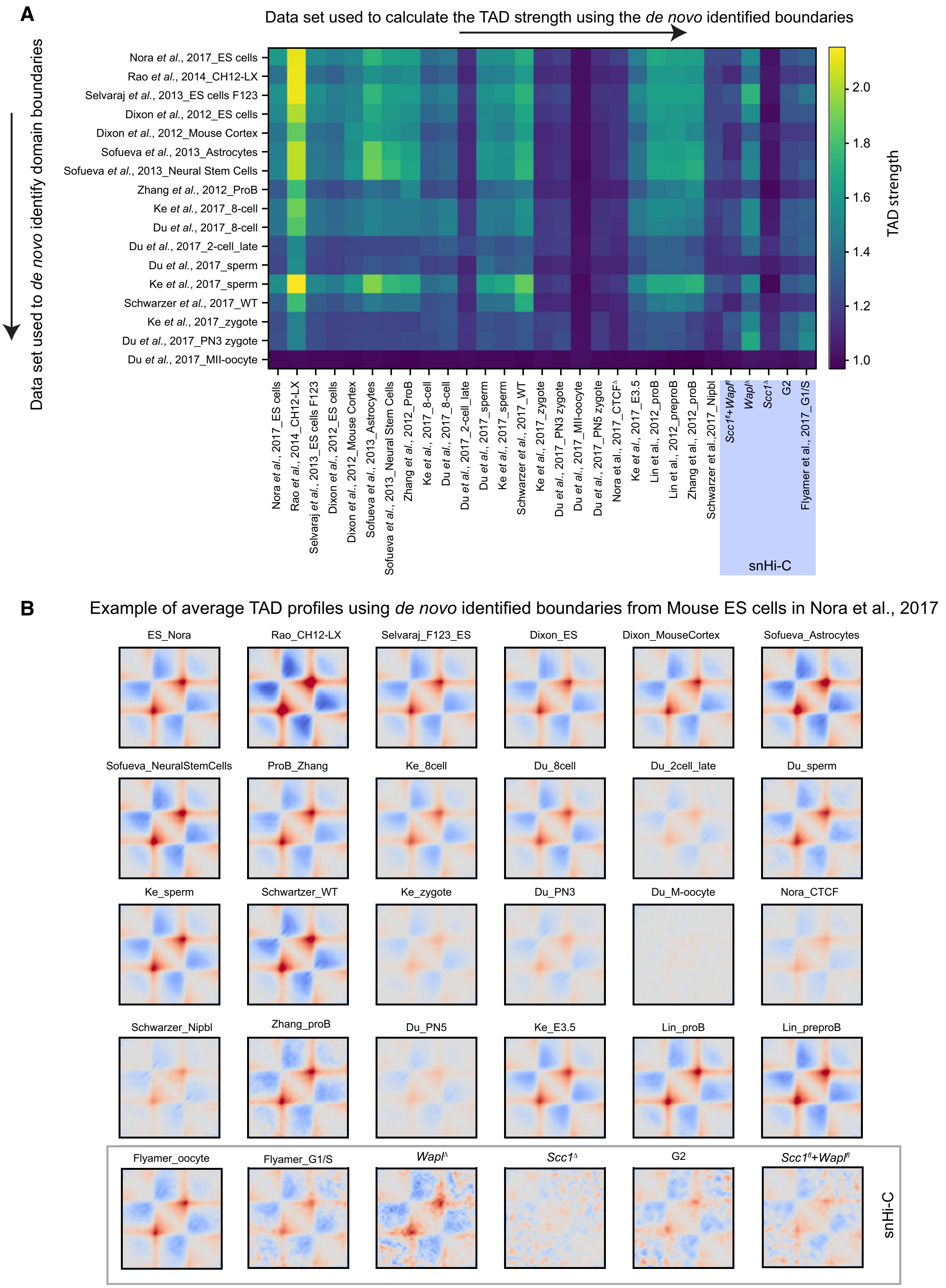

Figure EV1.

© 2017 The Authors

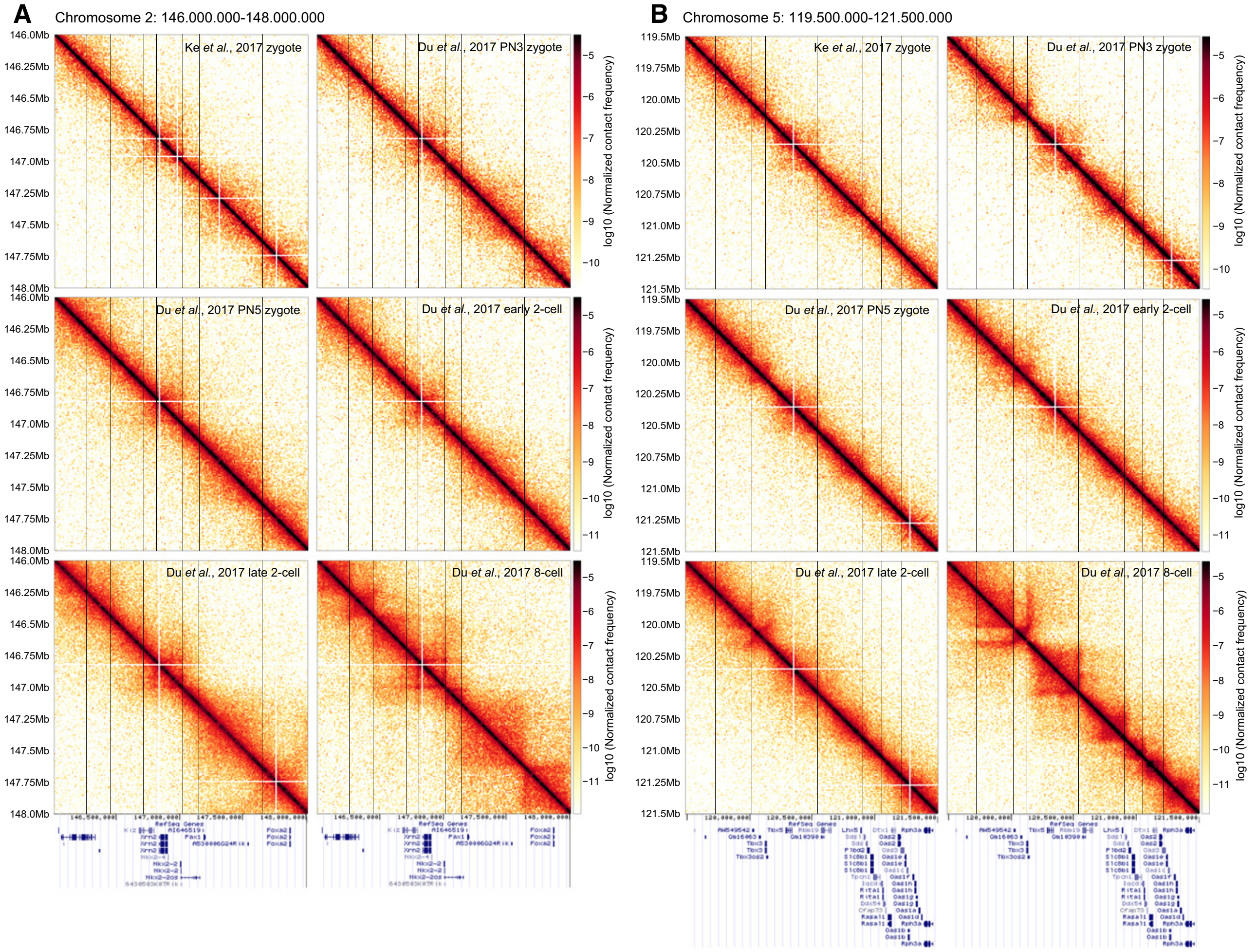

Figure EV2. TAD-like structures can be directly seen in zygote population Hi-C.

A An example region on chromosome 2 is shown for zygotic, two-cell, and eight-cell embryos, illustrating that in bulk Hi-C data (Du *et al*, 2017; Ke *et al*, 2017), it is possible to identify enrichments of contacts resembling TADs and loops. Vertical lines are drawn to guide the eye and compare the locations of boundaries visually identified in the Du *et al* (2017) eight-cell data. RefSeq gene annotation from the UCSC Genome Browser is shown below.

B The same as panel (A) but for an example region on chromosome 5.

© 2017 The Authors

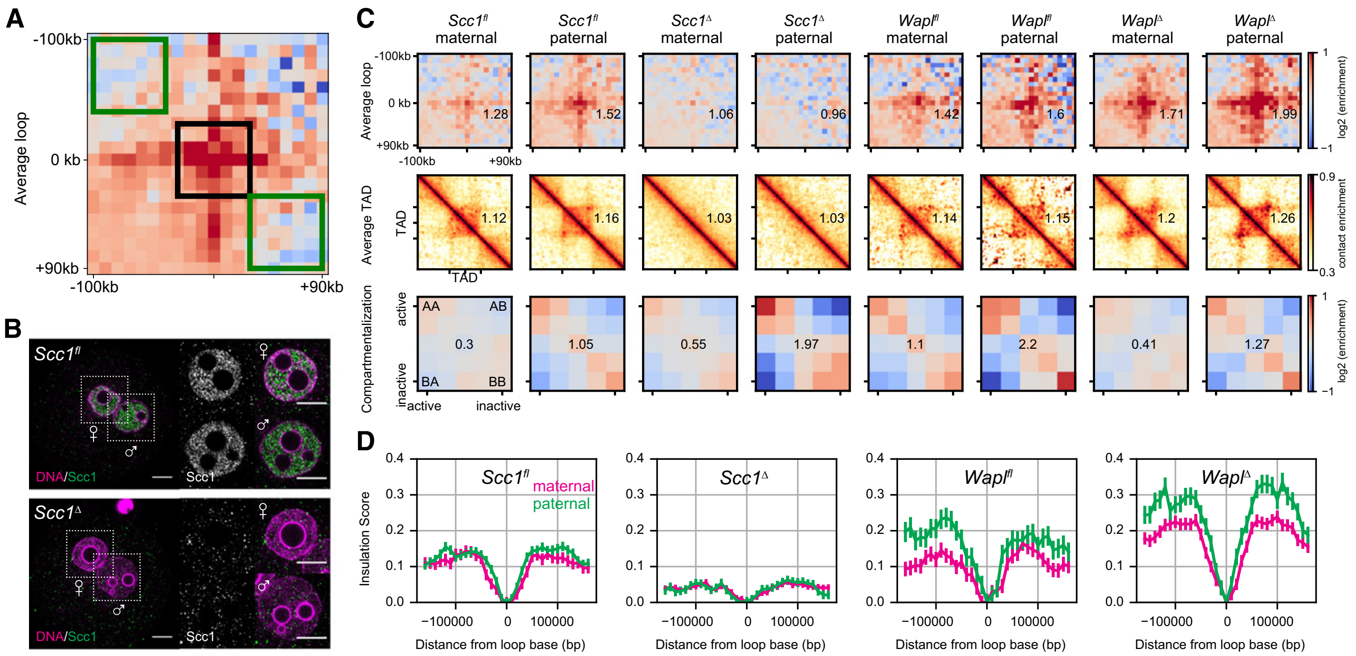

Figure EV3. Additional information on conditional knockouts.

A Loop strengths were calculated using the three 60 × 60 kb square regions shown. The average value within the middle box (black) was divided by the average of the combined top-left and bottom-right (green) boxes. The resulting number was subtracted by 1 to indicate the fractional increase in loop strength above the background.

B Immunofluorescence staining of Scc1 in *in situ* fixed *Scc1*^{fl} (n = 11) and *Scc1*$^{\Delta}$ zygotes (n = 12, from one experiment using two females of each genotype). DNA in magenta; Scc1 in gray/green. Images were adjusted in brightness/contrast in the individual channels using ImageJ. Scale bars: 10 µm. Left: single z-slice of zygotes. Right: single z-slice of the maximum cross-sectional area of maternal and paternal nuclei. Cropped area is indicated.

C Loops, TADs, and compartment saddle plots for the *Scc1*^{fl}, *Wapl*^{fl}, *Scc1*$^{\Delta}$, and *WaplΔ* conditions are shown separately for the maternal and paternal data. The average strength of each feature is indicated in each panel. Data shown are based on n(*Wapl*^{fl}, maternal) = 7, n(*Wapl*^{fl}, paternal) = 6, n(*WaplΔ*, maternal) = 8, n(*WaplΔ*, paternal) = 7, n(*Scc1*^{fl}, maternal) = 13, n(*Scc1*^{fl}, paternal) = 17, n(*Scc1*$^{\Delta}$) = 28, and n(*Scc1*$^{\Delta}$) = 17 nuclei, from at least two independent experiments using two to three females per genotype each.

D Insulation scores calculated with a sliding diamond of size 40 kb, with the “zero” position denoting a domain boundary identified previously in CH12-LX cells (Rao *et al*, 2014). Distances are reported in base pairs from a domain boundary. The average over all domain boundaries is reported; error bars are the standard error on the mean insulation score.

Source data are available online for this figure.

© 2017 The Authors

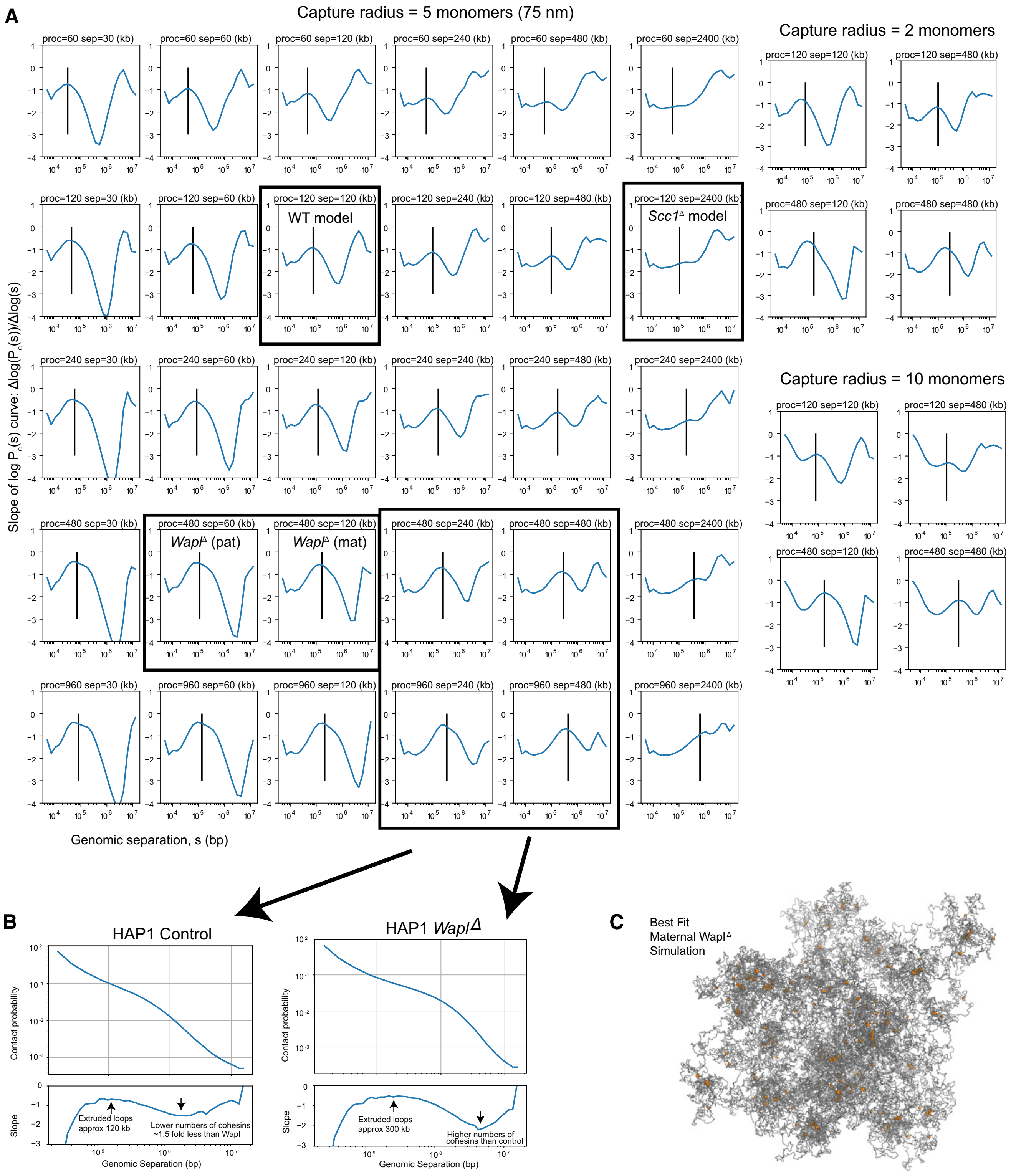
A
Capture radius = 5 monomers (75 nm)
proc=60 sep=30 (kb)
proc=60 sep=60 (kb)
proc=60 sep=120 (kb)
proc=60 sep=240 (kb)
proc=60 sep=480 (kb)
proc=60 sep=2400 (kb)
proc=120 sep=30 (kb)
proc=120 sep=60 (kb)
proc=120 sep=120 (kb)
WT model
proc=120 sep=240 (kb)
proc=120 sep=480 (kb)
proc=120 sep=2400 (kb)
Scc1Δ model
proc=240 sep=30 (kb)
proc=240 sep=60 (kb)
proc=240 sep=120 (kb)
proc=240 sep=240 (kb)
proc=240 sep=480 (kb)
proc=240 sep=2400 (kb)
proc=480 sep=30 (kb)
proc=480 sep=60 (kb)
WaplΔ (pat)
proc=480 sep=120 (kb)
WaplΔ (mat)
proc=480 sep=240 (kb)
proc=480 sep=480 (kb)
proc=480 sep=2400 (kb)
proc=960 sep=30 (kb)
proc=960 sep=60 (kb)
proc=960 sep=120 (kb)
proc=960 sep=240 (kb)
proc=960 sep=480 (kb)
proc=960 sep=2400 (kb)
Slope of log Pc(s) curve: Δlog(Pc(s))/Δlog(s)
Genomic separation, s (bp)
Capture radius = 2 monomers
proc=120 sep=120 (kb)
proc=120 sep=480 (kb)
proc=480 sep=120 (kb)
proc=480 sep=480 (kb)
Capture radius = 10 monomers
proc=120 sep=120 (kb)
proc=120 sep=480 (kb)
proc=480 sep=120 (kb)
proc=480 sep=480 (kb)
B
HAP1 Control
Contact probability
Slope
Extruded loops approx 120 kb
Lower numbers of cohesins ~1.5 fold less than Wapl
Genomic Separation (bp)
HAP1 WaplΔ
Contact probability
Slope
Extruded loops approx 300 kb
Higher numbers of cohesins than control
Genomic Separation (bp)
C
Best Fit Maternal WaplΔ Simulation

Figure EV4.

Figure EV4. Genome-wide contact probability, $P_c(s)$, for chromatin loci separated by genomic distances, s, underlying maternal *Wapl*$^{\Delta}$ simulation.

A Slopes of $P_c(s)$ curves as a function of genomic separation for polymer models of N = 30,000 monomers with loop extrusion. The rows show different loop extrusion processivities (proc), and columns show different linear separations (sep) between cohesins; the latter is related to the number of cohesins via the relation: separations = (chromosome length)/(number of bound cohesins). The vertical line on each plot indicates the average extruded loop length. All $P_c(s)$ plots in the left six rows/columns were calculated for a Hi-C contact radius of 5 monomers (75 nm). Plots on the right are a subset of plots on the left, for contact radius of 2 monomers (30 nm) and 10 monomers (150 nm), indicating that the inferred average extruded loop length does not vary significantly with the choice of Hi-C capture radius. Note that average extruded loop length is different from processivity, especially in a dense regime where processivity is greater than separation; due to stalling of cohesins when encountering each other and at simulated TAD boundaries, the average loop length then becomes less than processivity; see Goloborodko *et al* (2016) for details.

B The analysis of the slope of $\log(P_c(s))$ applied on recently published *Wapl*$^{\Delta}$ Hi-C data (see Haarhuis *et al*, 2017). Consistently with experimental FRAP data (Haarhuis *et al*, 2017), we find that in *Wapl*$^{\Delta}$ conditions, the processivity, which is linearly related to the chromatin-bound lifetime of cohesin, is increased $>$ 2 above control conditions. Similarly, we find that the number of bound cohesins is $>$ 1 but less than twofold enriched above controls in *Wapl*$^{\Delta}$; this is consistent with quantitative immunofluorescence data, showing a 1.5-fold enrichment for cohesins in *Wapl*$^{\Delta}$ versus controls (Haarhuis *et al*, 2017).

C A representative image of the maternal *Wapl*$^{\Delta}$ simulation. The chromatin fiber is colored in gray, and the locations of the cohesins are colored in orange, indicating that some cohesin vermicelli is visibly formed.

Source data are available online for this figure.

 © 2017 The Authors
