# Supplementary material for: A mechanism of cohesin‐dependent loop extrusion organizes zygotic genome architecture
Source: EMBO J. 2017 Dec 7;36(24):3600–18. doi: 10.15252/embj.201798083 (PMC5730859; doi:10.15252/embj.201798083)
Supplement: Supplementary file 5 — Movie EV1 [file EMBJ-36-3600-s005.zip › EMBOJ_98083R1_Movie_legend_EV1.docx]

**Movie EV1: Live-cell imaging of vermicelli formation in wildtype zygotes expressing Scc1-EGFP and H2B-mCherry**

Movie of live wild-type zygotes expressing Scc1-EGFP and H2B-mCherry (n=4 zygotes, from one experiment using two females). Individual imaging channels of the movie were adjusted in brightness/contrast. Scale bar: 10 µm. Hours after start of IVF are given.
